# Computer-Aided Reengineering towards Plastic Part Failure Minimization

**DOI:** 10.3390/ma14216303

**Published:** 2021-10-22

**Authors:** Tiago Pinho, Tatiana Zhiltsova, Mónica Oliveira, Andreia Costa

**Affiliations:** 1 Centre for Mechanical Technology and Automation (TEMA), Department of Mechanical Engineering, Campus Universitário de Santiago, University of Aveiro, 3810-193 Aveiro, Portugal; tp.spinho@ua.pt (T.P.); monica.oliveira@ua.pt (M.O.); 2 OLI-Sistemas Sanitários, S.A. Travessa de Milão, Esgueira, 3800-314 Aveiro, Portugal; andreiac@oli-world.com

**Keywords:** thermoplastics, mechanical behaviour, volumetric shrinkage, residual stress, designing for mouldability, numerical simulation

## Abstract

The work reported here intends to identify and mitigate the causes for failure in a plastic faucet holder, a part of an integral float faucet with a well-documented history of fracture occurrence. A methodology for the identification of hidden internal defects in plastic parts and the elaboration of the required corrective actions towards quality improvement is, therefore, presented. Firstly, part defects were identified via injection moulding process numerical simulation. The latter has enabled the prediction of an excessive volumetric shrinkage at the core of the faucet holder, highlighting the presence of internal voids and, hence, the possible deterioration of the load-bearing capacity. The supposition was later confirmed by X-ray topography scans. Part reengineering, consisting of localized thickness reduction, was the option chosen for decreasing the high shrinkage at the core. For validation purposes, structural analyses were carried out, with and without accounting for the injection moulding processing history. The results obtained during part structural analysis have enabled us to conclude that, when taking into account the residual stresses generated during injection moulding, the analysis more closely reflects the experimental data and allows us to implicitly envisage the propensity to fracture. Moreover, the part modifications, undertaken during the faucet holder reengineering, led to the reduction of the cumulative (processing and imposed by load) stresses by 50%, when compared to the original design analysed.

## 1. Introduction

Plastic components developed for engineering applications are usually intended for supporting mechanical efforts, which can vary significantly in magnitude. Thus, components’ design, defined at an early stage of the project, plays a critical role in the prediction of their expected mechanical performance and aesthetical appearance. Moreover, optimum design may lessen the environmental impact of the produced plastic components, through more rational use of raw materials and energy. An efficient design of a plastic part entails following up specific design for mouldability rules that guide the designer towards an optimum solution, comprising of shape, functionality, and processability [1,2].

Density and, hence, shrinkage variation, through the thickness of plastic components, are unavoidable in many cases, due to design requirements and restrictions. They are frequently reported as a source of internal and external defects, such as, warpage, voids and sink marks [3,4,5,6,7,8]. The latter, manifesting itself as depressions at the plastic component surface and appearing when the part surface is not solidified enough to prevent retraction from the mould wall, hence, constitutes an aesthetic defect [9]. Voids form when the melt solidified without pressure and plastic at the surface is rigid enough to prevent retraction. The voids, being undetectable by visual observation, may exert the most detrimental effect on the mechanical performance of injection-moulded components, weakening their capacity to withstand the loads while in service and leading to crack initiation and failure [10,11,12]. The influence of the excessive shrinkage, in chunky plastic parts moulded from fiber reinforced composites, was highlighted as the main cause of the occurrence of voids and warpage by Kleindel et al. [13]. Their conclusions were corroborated by the findings of Vaxman et al. for thick-walled, injection-moulded Noryl (polyphenylene-oxide/polystyrene) components [14].

In what concerns unfilled thermoplastics, the shrinkage is even more unconstrained than for reinforced composites. The life span of polypropylene hanger was drastically reduced by the presence of the voids, caused by excessive uncontrolled shrinkage [12].

The identification of the failure causes, in the majority of the above referred studies, were based on methodology, consisting of destructive and non-destructive experimental defect analysis, combined with the numerical simulation of the rheological and structural performance of the plastic components. However, concerning the pressure and thermally-induced residual stresses generated during injection moulding, none of these studies explicitly show their influence on the prediction of the structural performance. An exception was an approach used by Francis et al. [12], which emphasized the concentration of von Mises stress in the vicinity of the internal voids, explicitly modelled with the finite element mesh, according to the data obtained from destructive testing.

**Figure 1:**
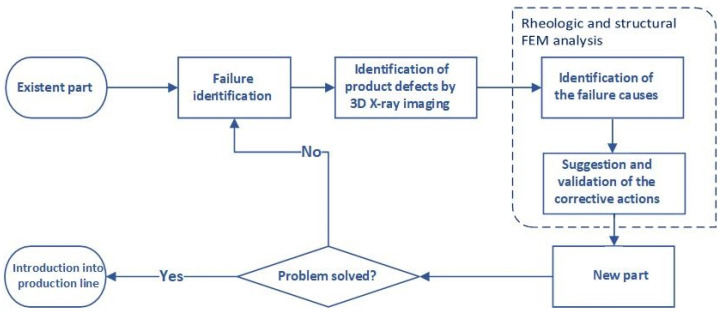
Flowchart of the reengineering process.

In light of the above, the objective of this study was to develop a framework for identification of the origins of failure in a plastic faucet holder, a component of float faucet assembly, and ameliorate its project. The reengineering was accomplished through the implementation of the design for mouldability concepts, assisted by FEM simulation of the float tap holder processing and its performance in service. The methodology, shown in Figure 1, will encompass three main phases:

- Identification of product defects and their probable causes with the aid of numerical simulation;
- Identification of product defects and their probable causes by 3D X-ray imaging;
- Proposed solutions and their evaluation.

The corrective actions focus on the redesign of the faucet holder, designated for the sake of brevity further in the text as (FH), based on the concepts of the design for mouldability and substantiated by the results of the rheologic and structural simulations, accounting for the residual stresses generated during processing. The implemented approach allows to assess the load-bearing capacity of the FH parts, as a function of the redesign and highlight the significance and influence of the residual stresses accumulated during processing on their mechanical strength.

## 2. Materials and Methods

### 2.1. Equipment and Materials

#### 2.1.1. Plastic Part

**Figure 2:**
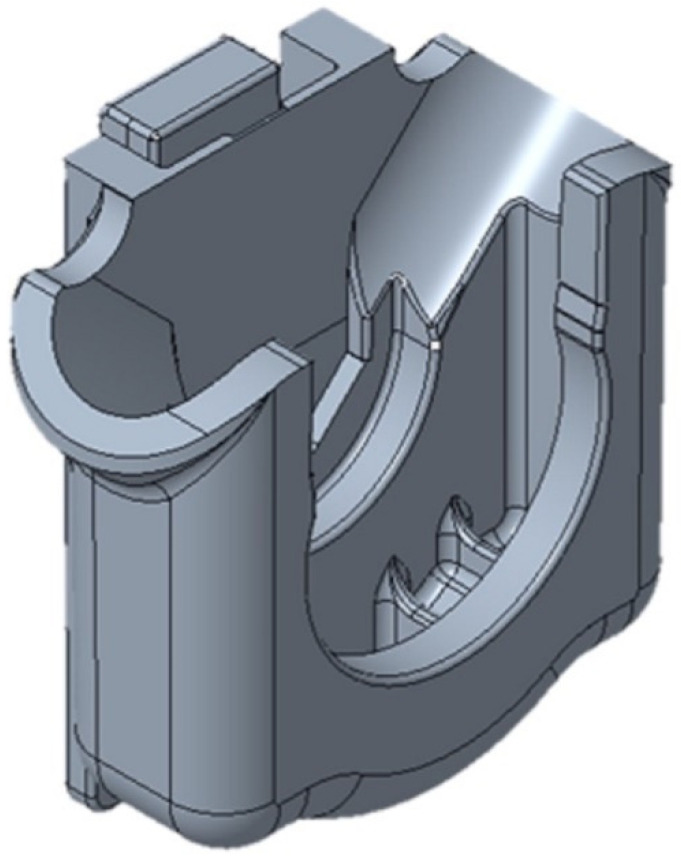
Faucet holder.

**Figure 3:**
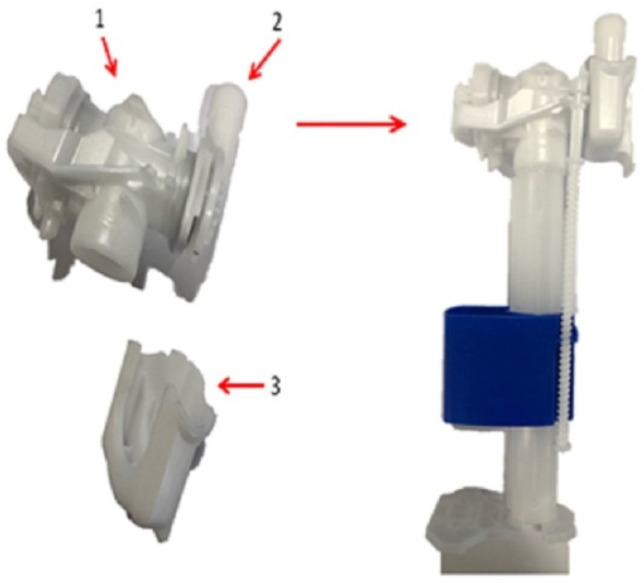
Float faucet assembly: 1-head of the float tap; 2-adapter to valve; 3-faucet holder.

The plastic part under investigation is a faucet holder (Figure 2), a component of the float faucet assembly (Figure 3), produced by OLI-Sistemas Sanitários S.A. As can be seen in Figure 3, the faucet holder (3) is mounted on the bottom of the adapter to valve (2), which, in turn, is connected to the head of the float tap (1). Afterwards, the assembly of these three components is mounted on the top of the float faucet assembly (right side of the Figure 3).

The function of the FH part is to support the float faucet, connected to the cistern, and to guarantee the perfect union and sealing between the head of the tap and the connecting element to the fitting. The FH has the overall dimensions of 52 mm of length, 52 mm of width, and 25 mm of depth and has a high thickness gradient along its length and width, where the thicker sections were designed to withstand the repeated mechanical stresses that occur when in service. FH parts were obtained by injection moulding and have a history of fracture failures, occurring during the quality control tests, which emulate the mechanical stresses taking place during service life (repeated flushing of the toilet cistern).

#### 2.1.2. Plastic Material, Injection Moulding Machine, and Processing Conditions

The FH parts were moulded with a hot runner, two-cavity mould, using a Victor Vs-80H (Victor Taichung Machinery Works Co., Ltd., Taichung, Taiwan) injection moulding machine, with a Ø36 mm injection screw, maximum injection rate of 135 cm^3^/s and maximum injection pressure of 196.7 MPa. The mould was equipped with copper beryllium alloy (AMPCO 83) inserts for more efficient heat removal from the deep cavities. Injection moulding processing conditions are presented in Table 1.

To withstand application of the repeated cyclical forces and comply with tight dimensional tolerances, FH parts were moulded from semi crystalline thermoplastic POM (HOSTAFORM C27021) by CELANESE(Dallas, TX, USA) with Melt Volume Rate (MVR) of 24 cm^3^/10 min (190 °C, 2.16 kg), pre-dried before processing for 3 h at 120 °C. Its thermo-physical and mechanical properties are presented in Table 2.

#### 2.1.3. Identification of the Defects

**Figure 4:**
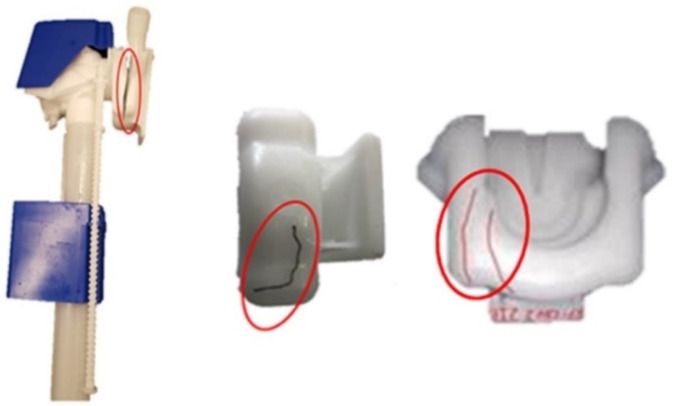
Fractured float tap holder (photograph kindly provided by OLI-Sistemas Sanitários, S.A.).

The assessment of the defects was carried by visual inspection of FH parts, which fractured during durability factory testing and in service at the lateral zones, as shown in Figure 4. Corrective actions, implemented at the shop floor for remediation of fracture occurrence, were essentially focused on the thickness increase at the problematic sections of FH parts towards strengthening their capacity to withstand mechanical solicitations. This intuitive approach, however, did not lead to fracture minimization. Moreover, as it has already been pointed out earlier, the localized thickness increase does not comply with the design for mouldability rules and may result in the further deterioration of FH parts quality.

#### 2.1.4. Assessment of the FH Parts Dimensional Tolerances

**Figure 5:**
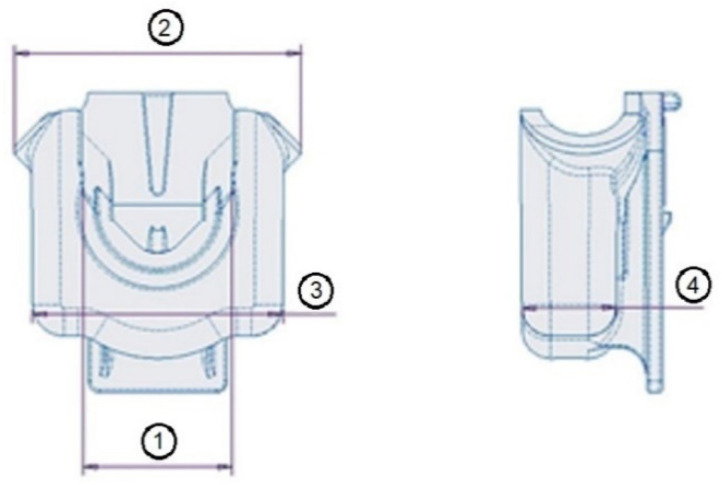
Identification of the location for dimensional measurements.

Considering that dimensional control is a critical parameter for the part under analysis, the dimensional tolerances in four directions, shown in Figure 5, were assessed. Statistical significance of the measurements was achieved by measuring 20 FH parts, using a digital caliper ABSOLUTE Digimatic Caliper Series 551 (instrument error ± 0.01 mm) from Mitutoyo America Corporation (Los Angeles, CA, USA). The mean value and error, associated with each of these dimensions, were calculated. These measurements, designated in Figure 5 as 1, 2, 3 and 4 will be referred further in the text as D.1, D.2, D.3, and D.4. and were used for validation of the numerical simulation results.

#### 2.1.5. Microscopy

3D X-ray imaging was conducted on the FH part to elucidate the presence of the internal defects impossible to identify by visual inspection. When exposed to X-ray radiation, the internal structure of the part will absorb it at different percentages, which leads to the identification of internal defects in the part. It was accomplished by using a Bruker MicroCt Skyscan 1275 microscope (Bruker Corporation, Billerica, MA, USA). A sequence of projections was obtained and reconstructed using NRECON software (Micro Photonics Inc., Allentown, PA, USA). CTAN software were used for 2D/3D image analysis and processing (Bruker Corporation, Billerica, MA, USA).

### 2.2. Modelling and Simulation of FH Processing and Performance

#### 2.2.1. FH Part Redesign

**Figure 6:**
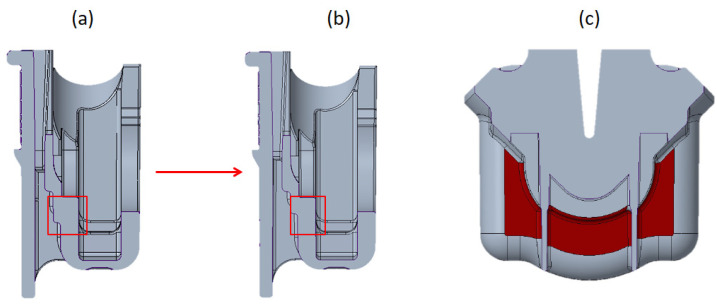
Redesign 1: (**a**) FH initial geometry cross section; (**b**) FH redesign 1 cross section; (**c**) localized thickness reduction.

Two redesign options were tested. Considering that the interior dimensions of the FH must not be altered, as it encompasses two other components of the float faucet assembly, the alterations were achieved by reducing the thickness inward the outer surface. The first option consisted in a reduction of 0.5 mm at the specific zones of the part, highlighted in Figure 6, where with the initial design and respective alteration of the cross section are shown, respectively, in the fragments (a), (b), and (c).

**Figure 7:**
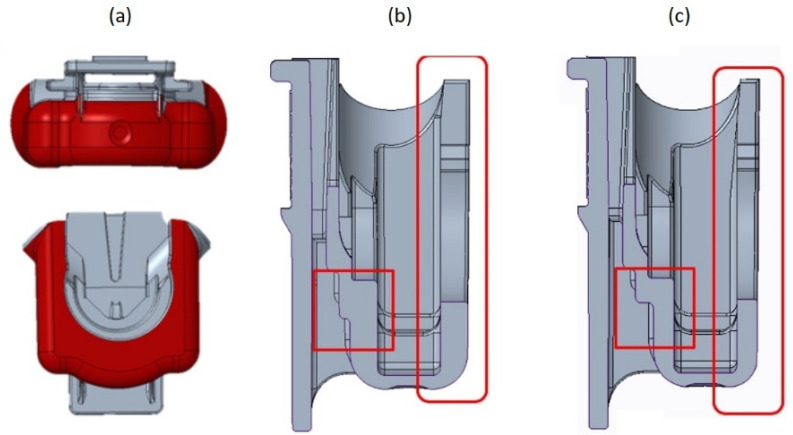
Redesign 2: (**a**) thickness reduction areas; (**b**) FH initial geometry cross section; (**c**) FH redesign 2 cross section.

Another option encompassed a more extensive alteration of FH thickness, both in extension and magnitude, which was accomplished by its reduction of 1 mm at the zones highlighted at fragment (a) of Figure 7, which resulted in more uniform thickness along the FH part, as can be verified from the fragment (b) and (c) of the same figure.

#### 2.2.2. Mesh Independence Study

To understand the influence of the FH parts design on the potential problems caused by processing, the modelling and numerical simulations of the injection moulding process were carried out with Autodesk Moldflow Insight (AMI) 2019 software (Autodesk, San Rafael, CA, USA). First, the FH part domain was discretized with 3D mesh of 10-node quadratic tetrahedral elements, the most suitable for models with complex shapes and significant thickness variation.

**Figure 8:**
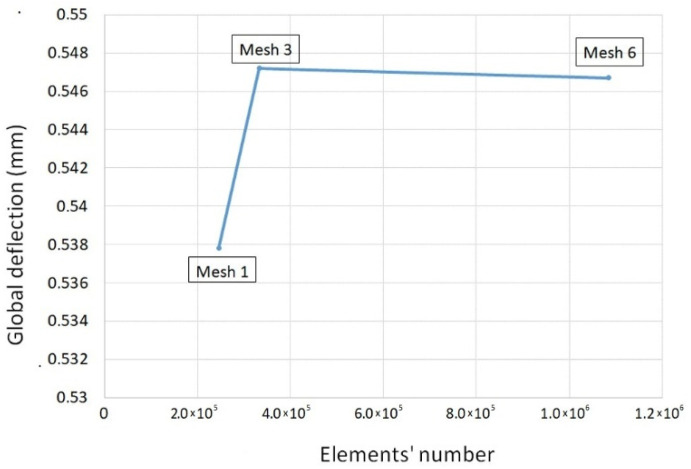
Predicted global deflection vs. mesh refinement.

To ensure that the obtained numerical solution results are invariant to the mesh refinement, the mesh independence study was executed by refining the global edge length of elements from 1.6 to 0.3 mm, along with the monitoring of their quality, expressed as maximum aspect ratio (lower the better). As shown in Table 3, more elements do not compulsory result in a better mesh quality. Based on this criterion, three types of mesh with different levels of refinement, designated as 1, 3, and 6 in Table 3, were subjected to analysis of filling, packing, and warpage, and the mesh influence on the simulation results was evaluated, in terms of global defection, as shown in Figure 8. Admissible variation of the numerical simulation results was established as 1%, and the mesh complying to this criterion was deemed to be the one with the optimum number of mesh elements.

The difference in the global part deflection between the less refined (mesh 1) and the most refined (mesh 6) mesh was in order of 1.65%. This difference was significantly reduced down to 0.09%, with an increase of the number of elements by 35%, from 245,650 (mesh 1) to 332,678 (mesh 3), making the latter a final choice for the subsequent simulations. It means that further refinements do not bring any benefits in precision but require more computational resources, as the difference in quantity of elements between the mesh 3 and 6 is three-fold.

Once the optimum mesh density was defined, the volume discretization of the redesigned models 1 and 2 were carried out with the mesh nº 3 refinement options (Table 3), resulting in 258,518 and 261,485 10-node quadratic tetrahedral elements, respectively.

#### 2.2.3. Injection Moulding Simulation of FH Parts

**Figure 9:**
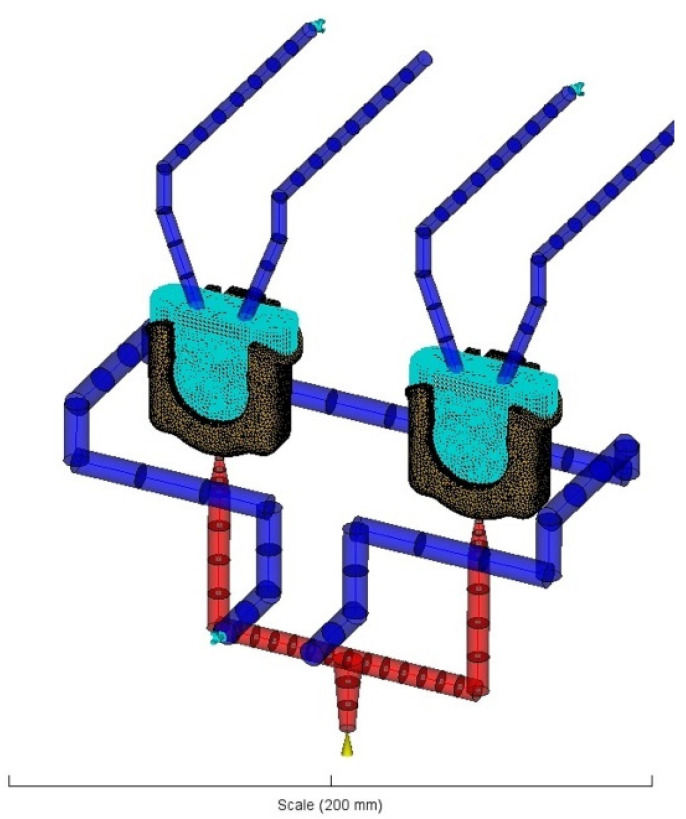
Layout of the FH parts and mould functional systems.

After the choice of the most suitable mesh, modelling of the FH parts, mould inserts, runner, and refrigeration systems were carried out to replicate, as closely as possible, their physical analogues (Figure 9). A diagnostic study of the FH parts processing was accomplished by the analysis sequence of cooling, filling, packing, and warpage, using plastic material, injection moulding machine, and processing conditions, described in detail in the Section 2.1.2 and Table 1. Redesigned FH models were also subjected to the same analysis sequence with the identical processing conditions and material in order to verify the influence of the modified designs on the issues identified during the diagnostic.

#### 2.2.4. Structural Simulation

During service, the FH parts are subjected to repeated loading, resulting from the high pressure at the toilet flushing system and, in order to be certified, are required to comply with specific directives. Therefore, it was of essence to submit the initial geometry of FH part and its redesigned versions 1 and 2 to a structural analysis.

Ansys 19.0 software (Canonsburg, Pennsylvania, USA) was used for Static Structural (SS) analysis of the initial design of FH part along with the redesign 1 and 2, based on the isotropic elasticity material model, providing accurate results until the material reaches the yield limit. This assumption will be verified by analysing the FH part deformation mode (elastic or plastic), when subjected to a typical operation.

**Figure 10:**
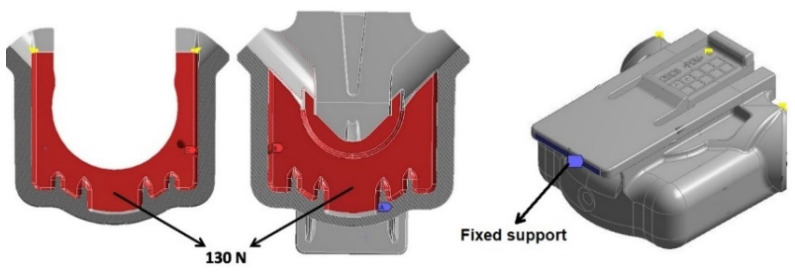
Layout of the FH parts.

The load applied during the static structural simulations was based on the pressure variation test, carried out at the testing facilities of OLI-Sistemas Sanitários S.A., in which the float faucet assembly is subjected to a maximum pressure of 3 (MPa). This pressure, imposed on the pipe, is in a direct contact with the internal walls of the FH part and exerts the mechanical stress on its internal walls. Considering the latter and the mounting position of the float faucet assembly inside the toilet flush system, the boundary conditions applied for the static structural analysis were force and fixed support, as demonstrated in Figure 10. The pressure was calculated according to the following equation:

F = (A × P)/2(1)

where, A = 86.59 (mm^2^) is a cross section of the pipe coupled inside the components assembled inside of the FH part; P = 3 is the pressure (MPa).

The original FH part and the redesigns 1 and 2 were meshed with the ten-node tetrahedral elements with the similar mesh density used in AMI simulations, resulting in 329,395, 329,155, and 323,319 elements, respectively. Two series of SS simulations were carried out, analysed, and compared. The first three simulations of the initial design of the FH part and its two redesigns were carried out, without accounting for the thermo-mechanical stresses that were induced during the injection moulding process. To verify the impact of the latter, the same sequence of the simulations was performed after mapping the residual stresses generated during processing and obtained from AMI rheologic simulations. It was accomplished with the aid of an auxiliary software HELIUS PFA 2019 (Autodesk, San Rafael, California, USA), which allows to map the residual stresses into the Ansys FEM models prior the SS analysis. More details of the mapping procedure may be consulted elsewhere [17].

Polymers, being ductile materials, are better represented by von Mises (maximum octahedral shear stress theory, which states that failure occurs when the maximum distortion energy (or maximum octahedral shear stress), at an arbitrary point in a stressed body, reaches the value equivalent to the maximum distortion at failure (yield) in simple tension and may be expressed, in terms of the principal components of stress as follows [16]:

(2) $$\sigma_{1}^{2}+\sigma_{2}^{2}+\sigma_{3}^{2}-(\sigma_{1}\sigma_{2}+\sigma_{2}\sigma_{3}+\sigma_{3}\sigma_{1})=\sigma_{f}^{2},$$

where *σ*_1_, *σ*_2_, and *σ*_3_ are principal stresses and *σ_f_* is von Mises equivalent stress.

## 3. Results and Discussion

### 3.1. Diagnostic of the Potential Problems in FH Parts

As it was mentioned above, the rheological simulations replicating the existent processing conditions of the FH part were carried out to identify the defects resultant from the injection moulding process and to gain an insight on their sources otherwise not assessable by visual observation. Therefore, the simulation results of importance, discussed here, are volumetric shrinkage, weld lines quality and prediction of the FH parts dimensions. The latter result was validated by comparison with the measurements performed on a batch of 20 FH parts.

**Figure 11:**
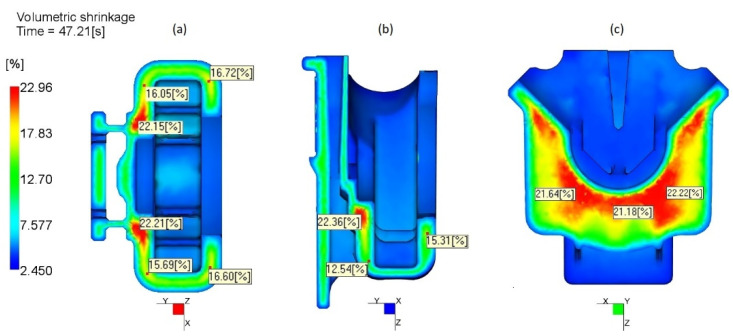
Volumetric shrinkage distribution through the thickness of the FH part: (**a**) cross-section 1, (**b**) cross-section 2, (**c**) cross-section 3.

For transversely isotropic materials as HOSTAFORM C27021, the guideline for volumetric shrinkage evaluation estimates its values to be equal to the shrinkage in the flow direction plus two times the shrinkage in the transverse direction (values of linear shrinkage obtained from Shrinkage Moulding Summary table of AMI 2019) [18]. A calculated value for HOSTAFORM C27021 was about 8.2%. This is only a rough reference, usually obtained by measuring shrinkage in parts of simple geometries with uniform thickness. For parts with high thickness gradient and complex configuration, volumetric shrinkage may deviate in both ways, as it becomes evident from Figure 11.

**Figure 12:**
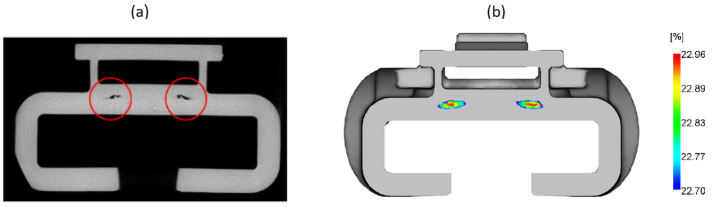
Location of the voids: (**a**) 3D X-ray slice of FH part, (**b**) Prediction of volumetric shrinkage at the void locations.

At thicker (4 mm) locations, the shrinkage of 23% was observed. This observation was corroborated by 3D X-ray tomography image (Figure 12a) where the voids were detected. The most probable cause for the voids’ occurrence is an excessive, noncontrolled shrinkage, as shown in Figure 12b, which may cause structural fragility and diminished load-bearing capacity. The presence of these voids leads to deterioration of the mechanical properties, making FH parts more susceptible to mechanical failures. The cause–effect relation between high thickness and subsequent non-uniform high shrinkage is well documented [11,19,20]. The latter and high shrinkage gradient (12–23%) at the core of the FH parts furthermore justify the redesign options towards the thickness reduction at these critical locations.

**Figure 13:**
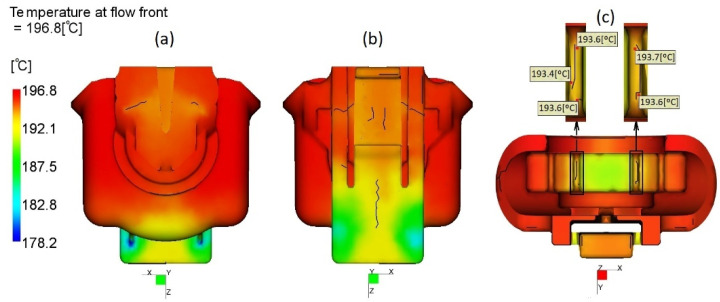
Flow front temperature at weld line locations: (**a**) front view, (**b**) back view, (**c**) top view.

Weld lines in FH parts were caused by flow patterns resultant from cavity configuration and have a potential to be their weakest points if the molten plastic temperature, at the moment of joining, is substantially cooler than the injection temperature [10,21]. A guideline to assure satisfactory weld quality is not let to drop the temperature of the melt at the weld line, as it forms below 20 °C below the injection temperature. Considering this factor, the weld lines severity and locations were assessed to understand how it may affect the mechanical resistance of FH parts. As shown in Figure 13, the quality of weld lines is satisfactory, at the melt temperature at the moments of the weld lines formation is about 2–6 °C higher than the injection temperature (190 °C), the latter being caused by shear heating of molten plastic during injection. Moreover, special attention was paid to assessment of the weld line quality at the ribs inside the FH part (Figure 13c) which are in contact with the adapter to valve (Figure 3) and, therefore, are subjected to additional mechanical solicitation during assembly and in service. At these critical locations, the predicted melt temperature was about 3, 4 °C above the injection temperature, indicating good weld lines quality, as well.

**Figure 14:**
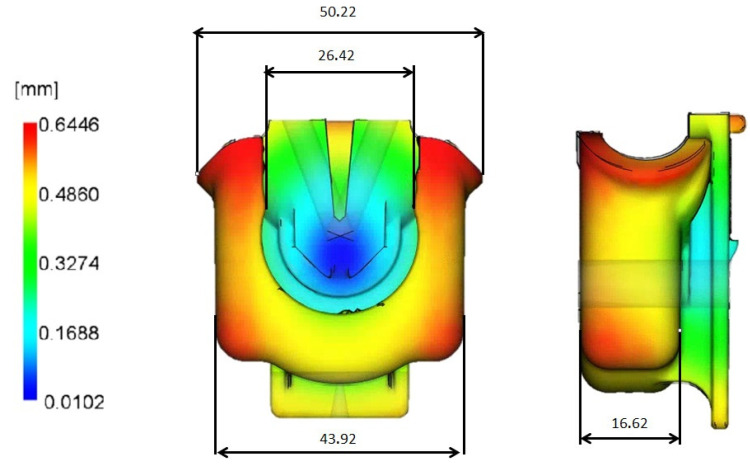
Numerically predicted FH part dimensions.

**Figure 15:**
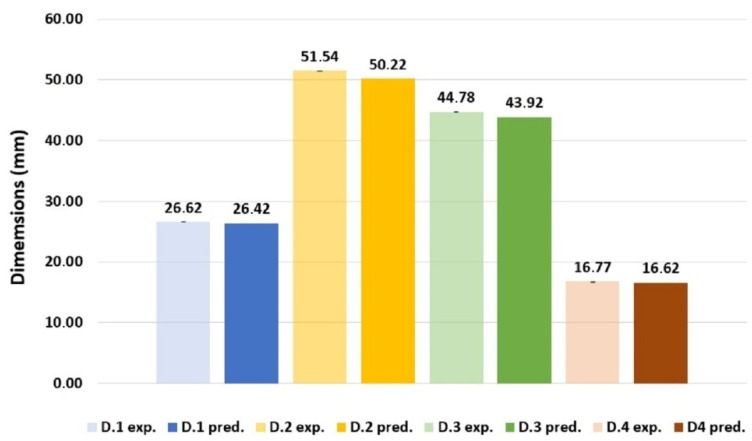
Comparison of experimental and predicted dimensions of the FH part.

Validation of the numerically predicted FH parts dimensions in four directions, as shown in Figure 14, was accomplished by comparison of the latter with the averaged values experimental measurements of 20 FH part at the same locations, displayed in Table 4 and Figure 15. Precision of the experimental dimension measurements was expressed quantitatively as the sample standard deviation of the observed values from repeated results under identical conditions and calculated as follows [22]:

(3) $$s=\sqrt{\frac{1}{n-1}\overset{n}{\underset{i=1}{\sum }}{\left(X_{i}-\bar{X}\right)}^{2}}$$

where $X_{i}$ is a sample value, $\bar{X}$ is a sample mean; and n is a count of the samples. It worth mentioning that sample standard deviation is low, making it barely detectable in Figure 15, which translates in good dimensional stability of FH parts along with representativeness of the sample size used for the dimensions’ assessment.

As shown in Table 4 and Figure 15, all predicted dimensions deviate very slightly from the experimental measurements. The maximum dimension variation of 2.56% was verified for the dimension D.2, while the lowest difference of 0.75% is on D.1, compared to the experimental mean values.

The hybrid diagnostic approach, conjugating both numerical and experimental procedures permitted to identify with a high degree of certainty that the main cause for fracture in FH parts is an excessive shrinkage of Hostaform C27021. This phenomenon, widely reported in the literature [3,14,19,23,24] is the main reason for occurrence of voids in thicker (4 mm) sections and hence weakening its load-bearing capacity.

### 3.2. Influence of the Redesigns on the Probability of Fracture

#### 3.2.1. Impact of the Design Modification on Volumetric Shrinkage

**Figure 16:**
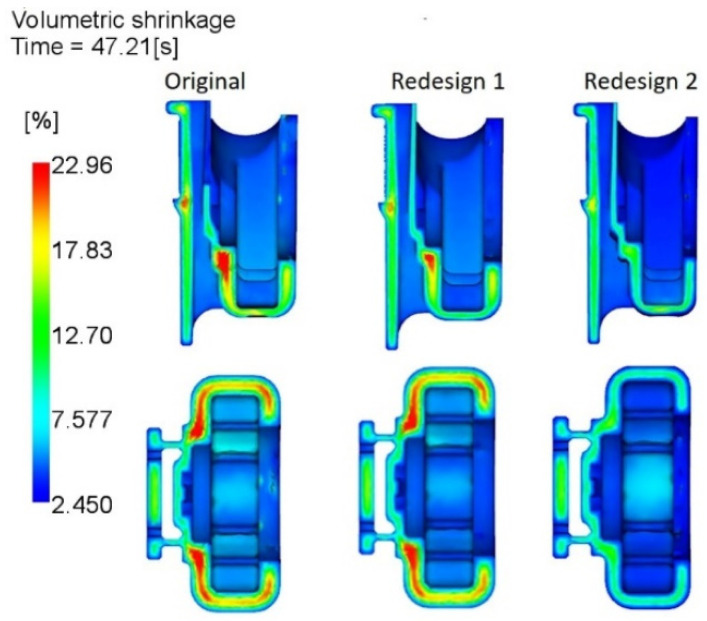
Comparison of the volumetric shrinkage variation in original geometry, redesigns 1 and 2.

As it can be seen from Figure 16, the redesign 1 with a reduction of 0.5 mm at specific zones (Figure 6), does not appear to have any significant influence on the shrinkage magnitude at the thickest core of the FH part. While with more extensive thickness reduction in the redesign 2 along the area under the load, it was possible to lessen the shrinkage variation in the core from 23% to about 12%. In what concerns the weld line severity and positions, highlighted during the diagnostics, no significant alteration was detected as the redesigns do not alter the flow pattern inside the cavity.

#### 3.2.2. Structural Simulation, without Accounting for Residual Stresses Generated during Processing

**Figure 17:**
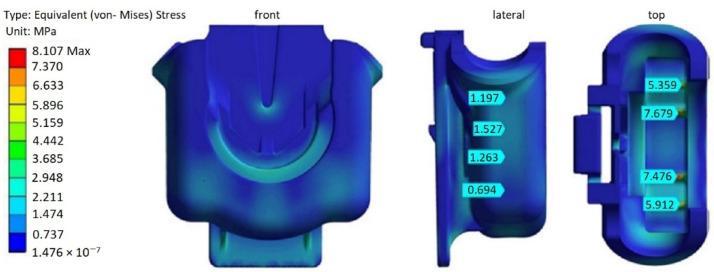
The von Mises stresses at the original FH part design (without processing history).

As shown in Figure 17, in the case of the original FH part design, the von Mises stresses, resulting from the imposed load are quite low varying for bulk of the part between 0.5 and 2 MPa. However, there are several zones located at the interior ribs where the stress concentration increase is more pronounced varying, according to the probe points depicted at Figure 17, between approximately 5.4 and 7.7 MPa, which may be attributed to the operation loads of FH parts combined with the reduced thickness of the ribs relatively to the overall thickness. Lateral zones, where the fracture have occurred at the previous model of the FH part (Figure 4), were also investigated in more details. However, the magnitude of the equivalent von Mises Stresses was quite low at these locations, below 2 MPa, which does not corroborate the experimental observation about propensity to failure there while in service.

**Figure 18:**
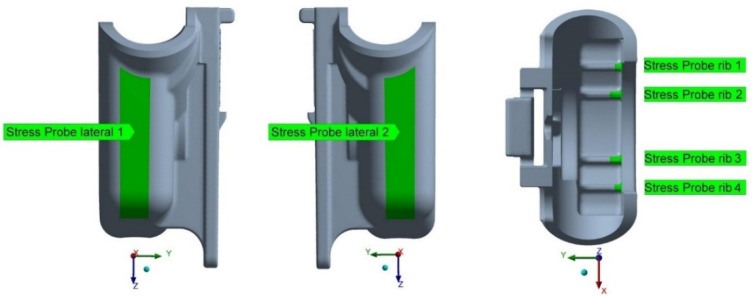
Locations of the von Mises stress probes.

To present the results in more orderly manner and allow for their more objective comparison, von Mises stresses probes, which retrieve the maximum value of the stress over the defined area, were implemented. The areas of interest were chosen at the internal ribs where the stress concentration was detected in the previous simulation results and at the lateral zones of the FH part, critical in terms of fracture occurrence, as show in Figure 18.

With the redesign option 1, there was almost no difference in von Mises stresses, compared to the original version of the FH part, at all locations, as can be verified from Table 5, which may be explained by the alteration of a very restricted part of the FH geometry (Figure 6). The maximum increase of about 2.15% in von Mises stresses was observed at rib 4. The more significant reduction in thickness, at redesign 2, led to a more pronounced increase in equivalent von Mises stresses, leading to their rise between 28–38%, at six locations under analysis (depicted in Figure 18), when compared to the original FH part design. However, it is worth recalling that the absolute values of the von Mises stresses are quite small and are well below 65 MPa, the yield stress limit of HOSTAFORM C27021, meaning that the deformations caused by the operation load are within the elastic boundaries and, thus, fully recoverable.

Nevertheless, the validity of these simulation results should be pondered with caution, as they do not account for polymer anisotropy, due to constrained quenching inherent to the injection moulding process. During the latter, the thermally- and pressure-induced residual stresses were developed, due to shrinkage during the cooling, coupled with the frozen layer growth with the varying pressure history. That is a reason that an assumption of the plastic material homogeneity may be not valid for the injection-moulded parts and, hence, may lead to the underestimated simulation results.

#### 3.2.3. Structural Simulation Accounting for Residual Stresses Generated during Processing

As previously referenced, the structural simulation, without considering the processing history of the part, may not be representative of real-life settings. To verify the impact of the residual stresses, induced during injection moulding on the mechanical performance of the FH parts, two more structural simulation analyses were carried out for the original version of the FH part and its redesign (option 2). The redesign option 1 was not considered, as it had very slight difference from the original model, and the expected results would be very similar to the latter, as it was previously corroborated by the volumetric shrinkage assessment (Figure 16) and the von Mises stresses prediction without accounting for the effect of processing (Table 5).

**Figure 19:**
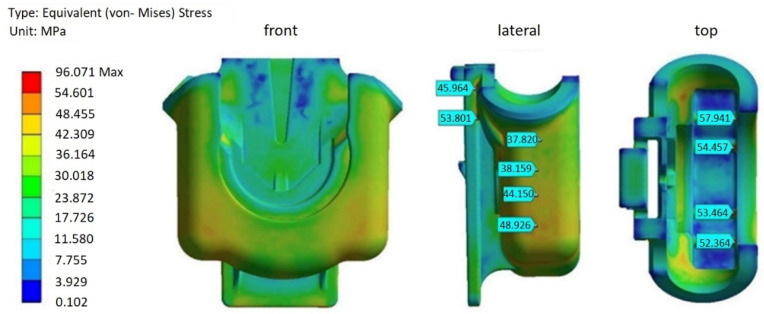
The von-Mises stresses: original design (with processing history).

**Figure 20:**
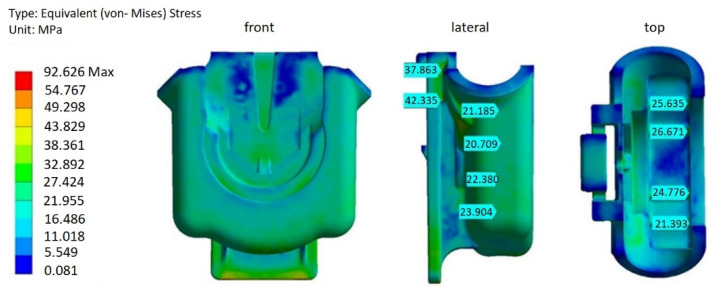
The von Mises stresses: redesign 2 (with processing history).

There was a considerable increase in von Mises equivalent stresses when the residual stresses, resultant from the injection moulding, were accounted for in the structural simulation for both the original and redesign 2 versions of the FH part, as shown in more detail in Figure 19 and Figure 20, and Table 6. For the part under consideration, it is a clear indication that the residual stresses, generated during processing, are more significant than the stresses originated by the mechanical load, which are imposed on the parts while in service.

The cumulative (loading and residual) stresses, as evident from the results of the original FH part (Figure 19), vary at the stress probe locations (Table 6), between 50 and 62 MPa. Taking a closer look at the lateral zones of the FH original model, it should be noted that the maximum values of the von Mises stresses are about 50 MPa.

These results, however, should be appraised with caution and may not be directly comparable to the yield limit of Hostaform C27021 (65 MPa) in absolute terms, but may serve, rather, as an indication of the residual stresses’ order of magnitude and distribution, in terms of geometry and thickness variations. The main reasons for these discrepancies are the assumptions applied in the AMI 3D anisotropic thermo-viscoelastic residual stress model, which assumes, for the sake simplicity, linear elastic behaviour in the solidified part and purely viscous behaviour in the melt [25]. Moreover, part-mould detachment, in-mould shrinkage, and post-moulding shrinkage, which result in more stress relaxation, are not accounted for in the model and, hence, may lead to some overestimation of the predicted residuals stresses. Slight discrepancies between the predicted and experimental results have been reported by several authors, emphasizing, nevertheless, the similar trend of the stress distribution with respect to the part thickness [26,27].

Considering the reasoning referred above, and assuming that the residual stresses may be overestimated to some degree, it is reasonable to conclude that the deformations are still in the elastic zone. Nevertheless, the probability of failure occurrence during the repetitive loading and unloading, while in service of the FH original model, may still be significant and can lead to part failure in a short period of time, as was documented in the past.

Comparing to the original FH part, the design 2 option with 1 mm reduction in thickness on most of the geometry, has undergone a reduction of about 50% in residual stresses, at all stress probe locations (Table 6), including the lateral zones. The latter reflects lessening and uniformization of the volumetric shrinkage (Figure 16), due to the reduction of the part thickness. This observation corroborates the results reported by several authors [8,11,28,29], which reported that shrinkage reduction and uniformization lead to the development of lower residual stresses. Moreover, by decreasing the residual stresses by half, superior performance of the FH parts should be expected under dynamic loading when in service.

In addition, it should be noted that maximum values of von Misses stress, for both of the original designs of the FH part (Figure 19 and Figure 20), are above 90 MPa. One of the reasons for this occurrence may be, as explained earlier, some overestimation of the residual stresses, due to the assumptions of the 3D AMI anisotropic thermo-viscous elastic residual stress model. Nevertheless, the high skewness of some mesh elements may also contribute to the overestimation of the residual stresses. It was reported in the literature that mesh skewness may adversely affect the accuracy of a numerical scheme, advising a maximum skewness of less than 0.9 [30,31]. These values are, however, very difficult to achieve without considerable mesh refinement, constrained by CPU time and computer resource limitations, especially critical for 3D models of complex shapes. In case of the 3D meshes generated for the structural models of the different FH part topologies, most of the elements were in an acceptable range, with the average skewness about 0.3. However, about one percent of the total amount of cells is highly distorted, with a skewness between 0.9 and 0.99.

## 4. Conclusions

In the present paper, the defects causing failure in a plastic float tap holder were investigated. The part was moulded from an unfilled grade of POM and was susceptible to fracture at the lateral zones during durability factory testing and in service.

Primarily, the diagnostic was carried out starting with injection moulding process numerical simulation, by replicating the existent injection moulding processing conditions from the shop floor. The numerical simulation results indicated very high volumetric shrinkage in the FH part core, well above the expected values for POM. Excessive shrinkage, as widely reported in literature, may be an indication of formation of voids at the thicker sections of the float tap holder and, as a consequence, may result in mechanical resistance decrease. The latter was further confirmed experimentally by an X-ray tomography scan. At the voids’ locations, the predicted volumetric shrinkage was the highest, reaching almost 23%.

Having, as an objective, the reduction of volumetric shrinkage at the core, two redesign options were considered. The redesign option 2, with 1 mm reduction in thickness on most of the geometry, was deemed to the be most efficient, as it led to the reduction in volumetric shrinkage in the core by half and, hence, to eventual minimization or elimination of voids.

Structural simulations, carried out to investigate how the original model and its modified version can withstand the typical operation conditions, revealed that the equivalent von Mises stresses were several times higher, for both the original and redesigned models, when, beside the loads imposed by operation, the residual stresses generated during injection moulding were accounted for. FH original model was under stress about 50 MPa at the lateral zones, where the fracture has taken place. Even considering that this value may be overestimated to some degree, these data, in conjunction with the history of failures, suggest that the FH original model will have a shorter life span and more propensity to fracture, when subjected to the cyclic loading and unloading while in service.

With a decrease in thickness, accomplished with redesign 2, the cumulative residual and working load stresses underwent a decrease of 50%, when compared to the original FH part, indicating more mechanical resistance under the same working conditions and, hence, higher durability.

The proposed methodology helps to identify the hidden internal defects in plastic parts and elaborate a set of corrective actions towards the improvement of their quality (after processing and in service). Moreover, this methodology, when applied at the early stage of the mould conception, may detect and correct the eventual processing problems at, eventually, no cost.

## Figures and Tables

**Table 1:** Injection moulding processing conditions.

Processing Condition	Value
Melt temperature (°C)	190
Cooling water temperature (°C)	80
Injection time (s)	1.6
Packing time (s)	16
Packing pressure (MPa)	28
Cooling time (s)	27

**Table 2:** Thermo-physical and mechanical properties of HOSTAFORM C27021 [15].

Density *Ρ* (kg/m^3^)	Coefficient of Thermal Expansion A (10^−6^/K)	Young’s Modulus E (MPa)	Poisson Coefficient ν	Bulk Modulus K * (MPa)	Shear Modulus G * (MPa)	Yield Stress $\sigma_{y}$ (MPa)	Tensile Strength $\sigma_{u}$ (Mpa)
1410	110	2900	0.4	4833	1036	65	55

* The values of the bulk and shear moduli were calculated according to the procedure described elsewhere [16].

**Table 3:** Mesh refinement variation.

Mesh nº	Global Edge Length	Max. Aspect Ratio	Elements’ Number
1	1.5	59	245,650
2	1.3	64	299,727
3	1.2	55	332,678
4	1.0	58	441,818
5	0.8	65	611,380
6	0.5	49	1,083,970

**Table 4:** Experimental and numerically predicted FH part dimensions.

	D.1 (mm)	D.2 (mm)	D.3 (mm)	D.4 (mm)
Predicted by AMI	26.42	50.22	43.92	16.62
Experimental average	26.62	51.54	44.78	16.77
Standard Deviation	0.0214	0.0295	0.0168	0.0207

**Table 5:** The von Mises stresses, without accounting for processing history.

Stress Probe Locations	*σ_or._* (MPa)	*σ*_*r*__1_ (MPa)	*σ*_*r*__2_ (MPa)	Δ*σ*_*vr*1_^1^ %	Δ*σ*_*vr*2_^2^ %
Lateral 1	1.62	1.61	2.15	0.62	33.54
Lateral 2	1.60	1.62	2.09	1.25	29.01
Rib 1	6.08	6.15	8.46	1.15	37.56
Rib 2	8.03	8.11	10.90	1.00	34.40
Rib 3	8.07	8.11	10.89	0.50	34.28
Rib 4	6.44	6.58	8.42	2.17	27.96

Δ*σ_vr_*_1_^1^—stress variation from the original design to the redesign 1. Δ*σ_vr_*_2_^2^—stress variation from the original design to the redesign 2.

**Table 6:** The von Mises stresses difference, with and without accounting for processing history.

Stress Probe Locations	*σ_or._* (MPa)	*σ_or.p.h._*^1^ (MPa)	*σ_r._*_2_ (MPa)	*σ_r._*_2*p.h.*_^2^ %	Δ*σ_p.h._* %
Lateral 1	1.62	52.61	2.15	27.79	47
Lateral 2	1.60	50.1	2.09	28.14	44
Rib 1	6.08	61.83	8.46	32.73	47
Rib 2	8.03	57.71	10.90	28.17	51
Rib 3	8.07	60.34	10.89	27.87	54
Rib 4	6.44	60.63	8.42	28.61	53

*σ_or.p.h._*^1^—The von Mises stress (original model with processing history). *σ_r._*_2_*_p.h._*^2^—The von Mises stress (redesign 2 with processing history).

## Data Availability

The data that support the findings of this study are available on request from the corresponding author.
